# High glucose promotes vascular smooth muscle cell proliferation by upregulating proto-oncogene serine/threonine-protein kinase Pim-1 expression

**DOI:** 10.18632/oncotarget.19368

**Published:** 2017-07-18

**Authors:** Keke Wang, Xiaojiang Deng, Zhihua Shen, Yanan Jia, Ranran Ding, Rujia Li, Xiaomin Liao, Sisi Wang, Yanping Ha, Yueqiong Kong, Yuyou Wu, Junli Guo, Wei Jie

**Affiliations:** ^1^ Department of Pathology, School of Basic medicine Sciences, Guangdong Medical University, Zhanjiang, P.R. China; ^2^ Department of Cardiovascular, Nanfang Hospital, Southern Medical University, Guangzhou, P.R. China; ^3^ Department of Pathology, Union Hospital, Tongji Medical College, Huazhong University of Science and Technology, Wuhan, P.R. China; ^4^ Cardiovascular Institute of 1st Affiliated Hospital & Key Laboratory of Tropical Diseases and Translational Medicine of Ministry of Education, Hainan Medical University, Haikou, P.R. China

**Keywords:** high glucose, vascular smooth muscle cell, Pim-1, cell proliferation, STAT3 signaling, Pathology Section

## Abstract

Serine/threonine kinase proviral integration site for Moloney murine leukemia virus 1 (Pim-1) plays an essential role in arterial wall cell proliferation and associated vascular diseases, including pulmonary arterial hypertension and aortic wall neointima formation. Here we tested a role of Pim-1 in high-glucose (HG)-mediated vascular smooth muscle cell (VSMC) proliferation. Pim-1 and proliferating cell nuclear antigen (PCNA) expression levels in arterial samples from streptozotocin-induced hyperglycemia rats were increased, compared with their weak expression in normoglycemic groups. In cultured rat VSMCs, HG led to transient Pim-1 expression decline, followed by sustained expression increase at both transcriptional and translational levels. Immunoblot analysis demonstrated that HG increased the expression of the 33-kDa isoform of Pim-1, but at much less extent to its 44-kDa plasma membrane isoform. D-glucose at a concentration of 25 mmol/L showed highest activity in stimulating Pim-1 expression. Both Pim-1 inhibitor quercetagetin and STAT3 inhibitor stattic significantly attenuated HG-induced VSMC proliferation and arrested cell cycle progression at the G1 phase. Quercetagetin showed no effect on Pim-1 expression but decreased the phosphorylated-Bad (T112)/Bad ratio in HG-treated VSMCs. However, stattic decreased phosphorylated-STAT3 (Y705) levels and caused transcriptional and translational down-regulation of Pim-1 in HG-treated VSMCs. Our findings suggest HG-mediated Pim-1 expression contributes to VSMC proliferation, which may be partly due to the activation of STAT3/Pim-1 signaling.

## INTRODUCTION

Vascular remodeling is a common pathological occurrence in diabetes mellitus (DM) that causes complications such as atherosclerosis, leading to enhanced patient morbidity and mortality [1]. As a prominent component of the vascular wall, smooth muscle cells play a crucial role in the initiation and propagation of diabetic vascular complications including abnormal cell growth in the atherosclerotic arterial intimae. Due to the complexity of the pathogenic mechanisms underlines the process of vascular remodeling in diabetic conditions [2, 3], to further identify new molecules involved in hyperglycemia/high-glucose (HG)-induced vascular smooth muscle cell (VSMC) proliferation, related signaling pathways warrant significant attention.

Proviral integration site for Moloney murine leukemia virus 1 (Pim-1) is a serine/threonine kinase first reported in hematological malignancies [4]. Pim-1 belongs to the Pim family, which also includes Pim-2 and Pim-3, and all members are well conserved among vertebrates [5]. Both the human and orthologous mouse genes have been reported to encode 33-kDa and 44-kDa isoform resulting from the use of alternative in-frame translation initiation codons[6]. In human cells, the subcellular localization of human 44-kDa Pim-1 is primarily on the plasma membrane, while the 33-kDa isoform is present in both the cytosol and nucleus. Pim-1 may promote proliferation in the nuclear compartment while its cytoplasmic localization may promote survival, implying different Pim-1 roles depending on cellular location [7]. Universally, Pim-1 is broadly expressed in various cancer cells [5, 8-10], in neurons [11], and in liver and spleen tissues [10, 12], as well as in some stem/progenitor cells [13, 14]. Regarding the cardiovascular system, expression of Pim-1 is reported in cardiomyocytes [7, 15], endothelial cells [16] and VSMCs [17-20]. With focus on VSMCs, Pim-1 expression was discovered in the VSMCs of balloon-injured rat carotid arteries and human coronary arteries [17], whereas the Pim-1-specific inhibitor, quercetagetin, or adenovirally introduced Pim-1 shRNA, markedly suppressed VSMC proliferation [17, 18]. Thus, aberrant Pim-1 expression plays a crucial role in diseases of vascular remodeling involving abnormal proliferation of VSMCs.

Previous studies on Pim-1-mediated proliferation signaling pathways in different cell types highlight the complexity of pathogenic mechanisms. For example, Akt activation induces Pim-1 expression, which protects the infracted myocardium in mice [7]. Furthermore, Pim-1 overexpression promotes cell survival by mediating the activation of Bcl-2 and Bcl-XL, as well as phosphorylation of Bad [7, 21]. In hematologic malignancies, signal transducer and activator of transcription 3/5 (STAT3/5), nuclear factor kappa-light-chain-enhancer of activated B cells (NF-kB) or HOXA9 can transcriptionally activate Pim-1, supporting cellular proliferation through modification of cell cycle regulators, such as cooperating with c-Myc and c-Myb, enhancing Cdc25A and Cdc25C, and inactivating p21 and p27 [22]. STAT family members include seven transcription factors (STAT1, STAT2, STAT3, STAT4, STAT5A, STAT5B, and STAT6) that have multiple roles in cell proliferation and survival [23]. STAT3 was originally identified as an acute-phase response factor activated by many cytokines, with an important role in the development of cardiovascular diseases, including pulmonary arterial hypertension (PAH) [19, 24]. We hypothesized that aberrant STAT3/Pim-1 activation may contribute to vascular remodeling diseases such as DM-induced atherosclerosis.

Our previous study revealed abnormal proliferation of VSMCs in streptozotocin (STZ)-induced thoracic arteries of type I diabetic rats [25]. In the present study, we further sought to explore the role of aberrant Pim-1 expression in the proliferation of VSMCs in HG conditions. We found that significant upregulation of 33-kDa Pim-1 was detected in VSMCs from STZ-induced type I diabetic rat arteries *in vivo* and HG-treated VSMCs *in vitro*. Furthermore, inhibition of Pim-1 activity by quercetagetin significantly attenuated HG-induced VSMC growth, and more importantly HG-activated STAT3/Pim-1 signaling was found to contribute to VSMC proliferation. Our results provide novel insight into DM-induced vascular complications.

## RESULTS

### Pim-1 and PCNA upregulation and alteration of phenotype-related gene expression in arterial samples of STZ-induced type I diabetic rats

**Figure 1:**
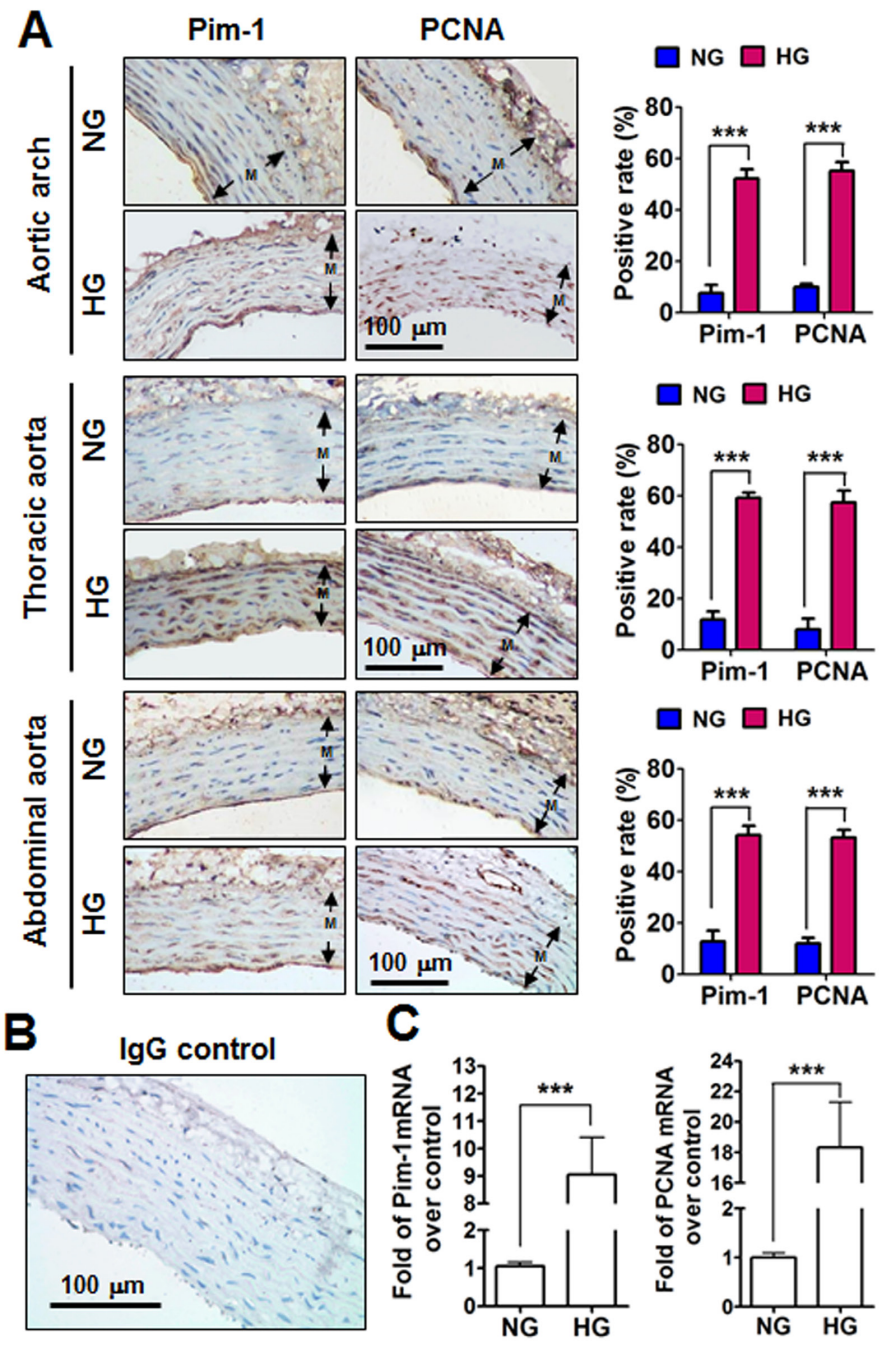
Pim-1 and PCNA expression in the tunica media (M) of the aortic arches, thoracic and abdominal arteries of STZ-induced hyperglycemia (HG) and normoglycemia (NG) rats **A.** Representative photographs of Pim-1 and PCNA protein expression in rat aortic arches, thoracic and abdominal arteries. Immunohistochemical analysis was used to detect Pim-1 and PCNA protein expression in the tunica media of arteries. Antigens were localized by DAB and tissues were counterstained with hematoxylin. Positive rates for Pim-1 and PCNA expression were calculated using at least three representative visions (200×) for each arterial sample. Both Pim-1 and PCNA were localized in the cytoplasm and nucleus. **B**. Non-immune IgG was used as a negative control. Original magnification: 200×, Bar = 100 μm. **C.** Pim-1 and PCNA mRNA expression in the tunica media of thoracic arteries were examined by qRT-PCR. NG, Normoglycemia (*n* = 7); HG, Hyperglycemia (*n* = 10).

Rats with a blood glucose level >/= 16.6 mmol/L were considered HG after intraperitoneal injection with STZ, while rats intraperitoneally injected with sodium citrate with normal blood glucose concentrations were served as normorglycemia (NG). Three weeks later, all rats were sacrificed and aortic arch, thoracic aorta and abdominal aorta were subjected to analyze of Pim-1 and PCNA expression. Immunohistochemistry showed weak expression of Pim-1 and PCNA protein in the VSMCs of NG rats; however, Pim-1 and PCNA protein expression levels were significantly enhanced in arterial-derived VSMCs from HG rats, and the positive signs of Pim-1 were in both nucleus and cytoplasm (Figure 1A). Immunofluorescence staining conformed that Pim-1 was co-located with α-SMA in the tunica media of thoracic aorta, which indicated that it was the VSMCs express Pim-1 protein (Supplementary Figure S1). Furthermore, HG clearly induced Pim-1 and PCNA mRNA expression compared with NG (Figure 1C). Additionally, mRNA levels of contractile markers of VSMCs such as SM22α (smooth muscle protein 22 alpha) and FHL2 (four and a half LIM domains 2) and myocardin (a nuclear factor that mediates part VSMC contractile markers) were reduced, while synthetic markers of VSMCs such as osteopontin, matrix metalloproteinase -2, -9 (MMP-2, MMP-9) were enhanced in HG rats (Supplementary Figure S2). These results indicated that 3-weeks of HG significantly induced the VSMC contractile phenotype to change into a synthetic phenotype in which Pim-1 upregulation and cell proliferation occurred.

### Upregulation of Pim-1 in HG-treated primary VSMCs *in vitro*

**Figure 2:**
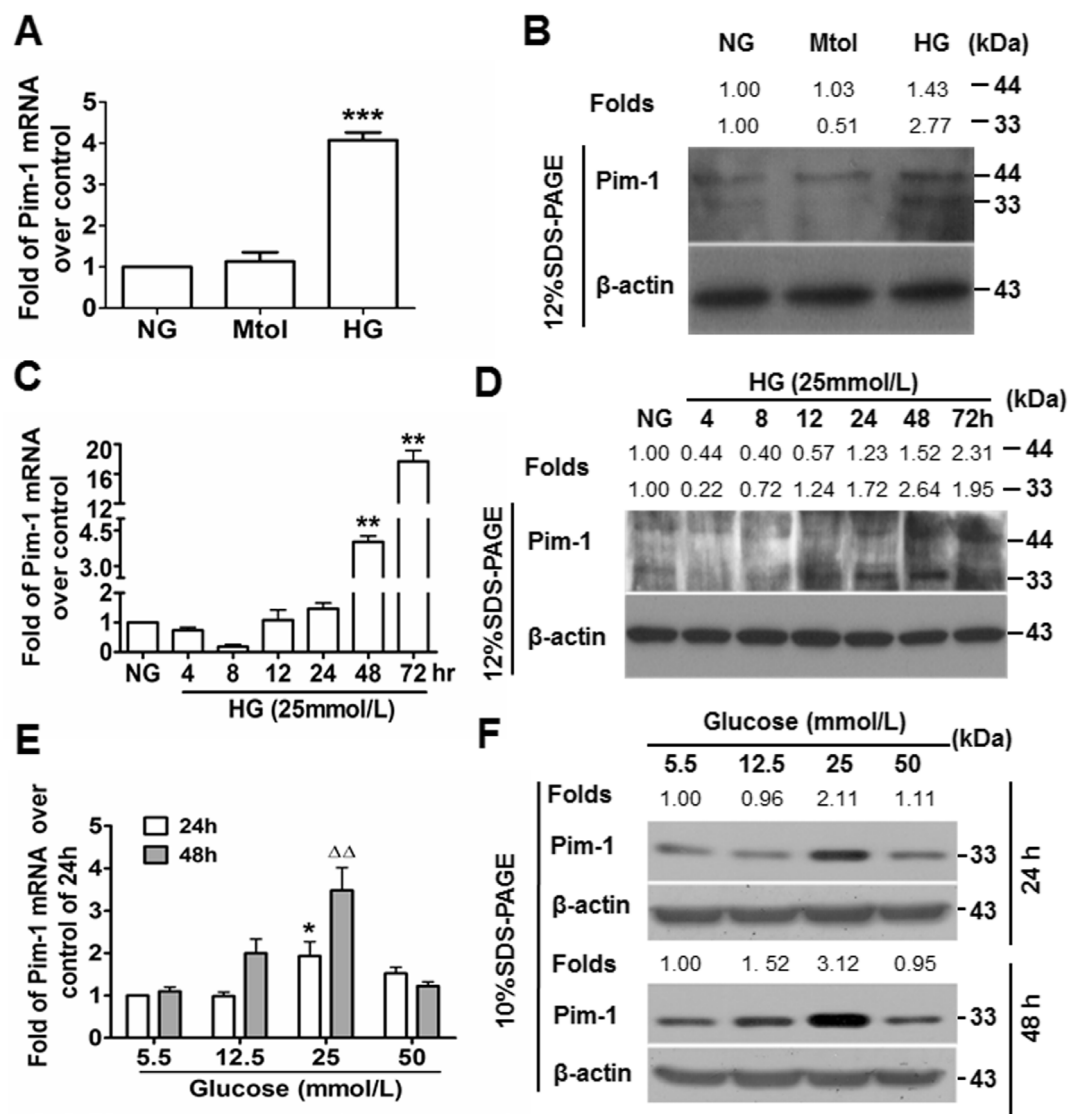
High glucose (HG) induces Pim-1 expression in cultured VSMCs **A.** qRT-PCR and **B.** western blotting were used to detect Pim-1 expression in VSMCs maintained in media supplemented with normal glucose (NG, 5.5 mmol/L D-glucose), Mtol (25 mmol/L D-mannitol) and high glucose (HG, 25 mmol/L D-glucose) for 48 hours. ***P < 0.001 *vs.* NG or Mtol. **C.** qRT-PCR and **D.** western blotting were used to detect Pim-1 expression in VSMCs maintained in NG (5.5 mmol/L D-glucose, 24 hours) and HG (25 mmol/L D-glucose, 4, 8, 12, 24, 48,72 hours), **P < 0.01 *vs.* NG. **E.** qRT-PCR and **F.** western blotting were used to detect Pim-1 expression in VSMCs maintained in media supplemented with various concentration of D-glucose (5.5, 12.5, 25, and 50 mmol/L) for 24 or 48 hours, *P < 0.05 *vs.* 5.5 mmol/L D-glucose (NG). ^∆∆^*P* < 0.01 *vs.* 5.5 mmol/L D-glucose (NG). For the western blot, changes of Pim-1 isoform levels were depicted as folds over controls.

The Pim-1 gene encodes 33-kDa and 44-kDa isoforms. The role of HG in Pim-1 expression by VSMCs was explored *in vitro*. Primary VSMCs were treated in normal glucose (NG), mannitol (Mtol, osmotic control) and HG media, and 48 hours later, cells were analyzed for Pim-1 expression. Quantitative real time polymerase chain reaction (qRT-PCR) and western blotting showed that Mtol affected Pim-1 expression slightly when compared with NG (*P* > 0.05), while HG treatment led to moRE significant upregulation of the 33-kDa than the 44-kDa Pim-1 protein (Figures 2A & 2B). These results indicated that upregulation of Pim-1 was HG-dependent, and HG stimulation preferentially induced 33-kDa Pim-1 isoform expression. Furthermore, we found that HG induced Pim-1 expression in VSMCs in a manner of transient decline following sustained increase at both transcriptional and translational levels (Figure 2C-2D) and glucose concentration-dependent manner, with the optimal glucose concentration being 25 mmol/L (Figure 2E & 2F). Consistent with the *in vivo* trend, 48-hour of *in vitro* HG-treatment also led to upregulation of synthetic markers of VSMC (Supplementary Figure S3). Of notable, the bands for Pim-1 isoforms could be separated well using 12% SDS-PAGE; under 10% SDS-PAGE, the bands could not be separated well. Thus, Pim-1 bands within 10% SDS-PAGE represent the total proteins including two isoforms.

### Quercetagetin and stattic repress HG-induced VSMC proliferation *in vitro*

**Figure 3:**
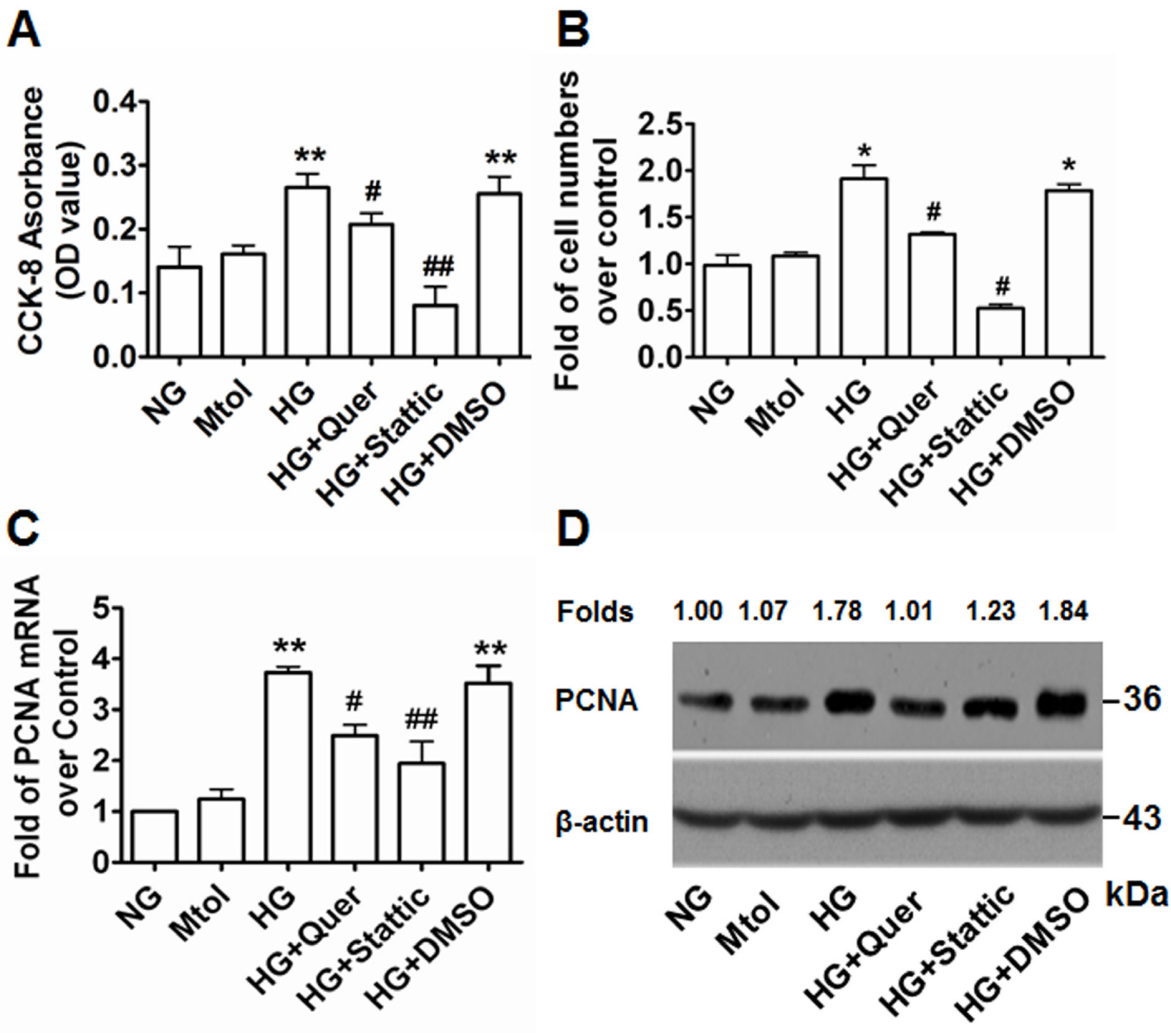
Quercetagetin and stattic attenuate HG-induced VSMC proliferation *in vitro* **A.** Cells (2 × 10^3^) were plated into 96-well plates and 6 hours later medium was changed to either NG (5.5 mmol/L D-glucose), Mtol (25 mmol/L D-mannitol), HG (25 mmol/L D-Glucose), HG+Quer (25 mmol/L D-Glucose + 5.5 μmmol/L quercetagetin), HG+Stattic (25 mmol/L D-Glucose + 10 μmol/L stattic), or HG+DMSO (25 mmol/L D-Glucose + 0.3% (v/v) DMSO) for 48 hours and the CCK-8 assay was used to detect cell viability. **P < 0.01 *vs*. NG or Mtol, ^#^*P* < 0.05 *vs*. HG or HG+DMSO, ^##^*P* < 0.01 *vs*. HG or HG+DMSO. **B.** Cells (5×10^5^) were plated into 6-well plates and 6 hours later the medium was changed as described above for an additional 48 hours and cell numbers were counted. *P < 0.05 *vs*. NG or Mtol; ^#^*P* < 0.05 *vs*. HG or HG+DMSO. **C.** qRT-PCR and **D.** western blotting were used to examine the expression of PCNA. **P < 0.01 *vs*. NG or Mtol; ^#^*P* < 0.05 *vs*. HG or HG+DMSO, ^##^*P* < 0.01 *vs*. HG or HG+DMSO. Quer, quercetagetin.

Accumulating evidence, including our own, has previously confirmed that exposure to HG condition contributes to VSMC proliferation [25-28]. To explore the relationship between Pim-1 expression and HG-induced VSMC proliferation, we investigated the contribution of Pim-1 inhibition on the proliferation of VSMCs exposed to HG. After 48 hours exposure to HG, cell viability was assessed using the cell counting kit-8 (CCK-8) assay. Cell numbers were counted and qRT-PCR and western blotting were used to examine expression of PCNA. As shown in Figure 3, exposure of VSMCs to HG led to significantly enhanced OD values, cell numbers and PCNA expression levels compared with NG and Mtol groups. This indicated that treatment of VSMCs with HG enhanced cell proliferation in an HG-dependent manner. Addition of the Pim-1 blocker quercetagetin (5.5 μmol/L) to the HG medium resulted in decreased cell viability, cell number and PCNA expression (Figure 3). Thus, HG-induced VSMC proliferation may be partly due to HG-induced Pim-1 upregulation and activity maintenance. Interestingly, we also observed that stattic (10 μmol/L) also blocked HG-induced VSMC proliferation, implying a crucial role for the activation of STAT3 signaling in HG-induced VSMC proliferation (Figure 3).

### Quercetagetin and stattic mainly arrest VSMC cell cycle at G1 phase

**Figure 4:**
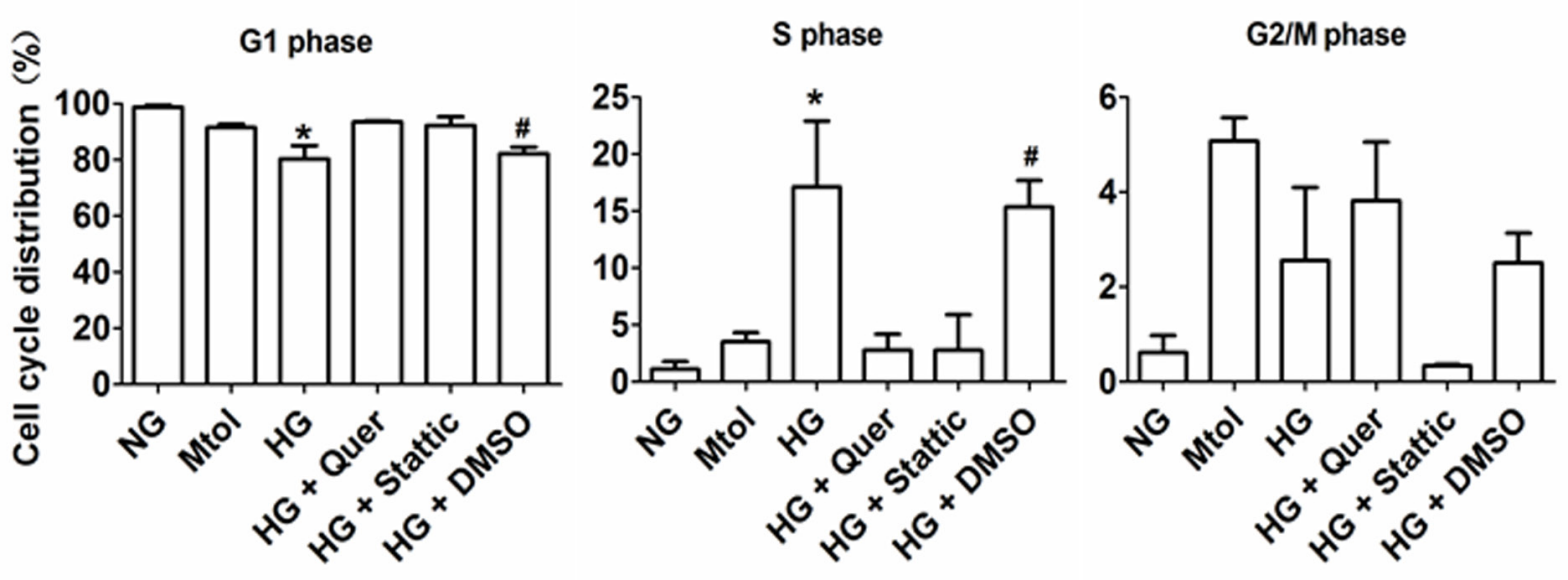
Cell cycle distribution of HG-treated VSMCs *in vitro* Data represent means SD from three independent experiments. HG, 25 mmol/L D-glucose. *P < 0.05 *vs*. NG, Mtol, HG+Quer or HG+Stattic. ^#^*P* < 0.05 *vs*. NG, Mtol, HG+Quer or HG+Stattic.

VSMCs were serum-starved for 12 hours, then exposed to various treatments. Flow cytometric analysis showed that HG stimulated G1 to S phase progression to a greater extent compared with NG and Mtol groups. Furthermore, administration of quercatagetin (5.5 μmol/L) significantly repressed HG-induced G1 to S phase cell cycle progression, indicating that quercatagetin inhibited cell proliferation by suppressing DNA synthesis in the cells. Additionally, administration of stattic (10 μmol/L) elicited inhibitory effects on HG-induced VSMC accumulation in S phase (Figure 4).

### Quercetagetin inhibits Pim-1 protein activity but not gene expression in HG-treated VSMCs

**Figure 5:**
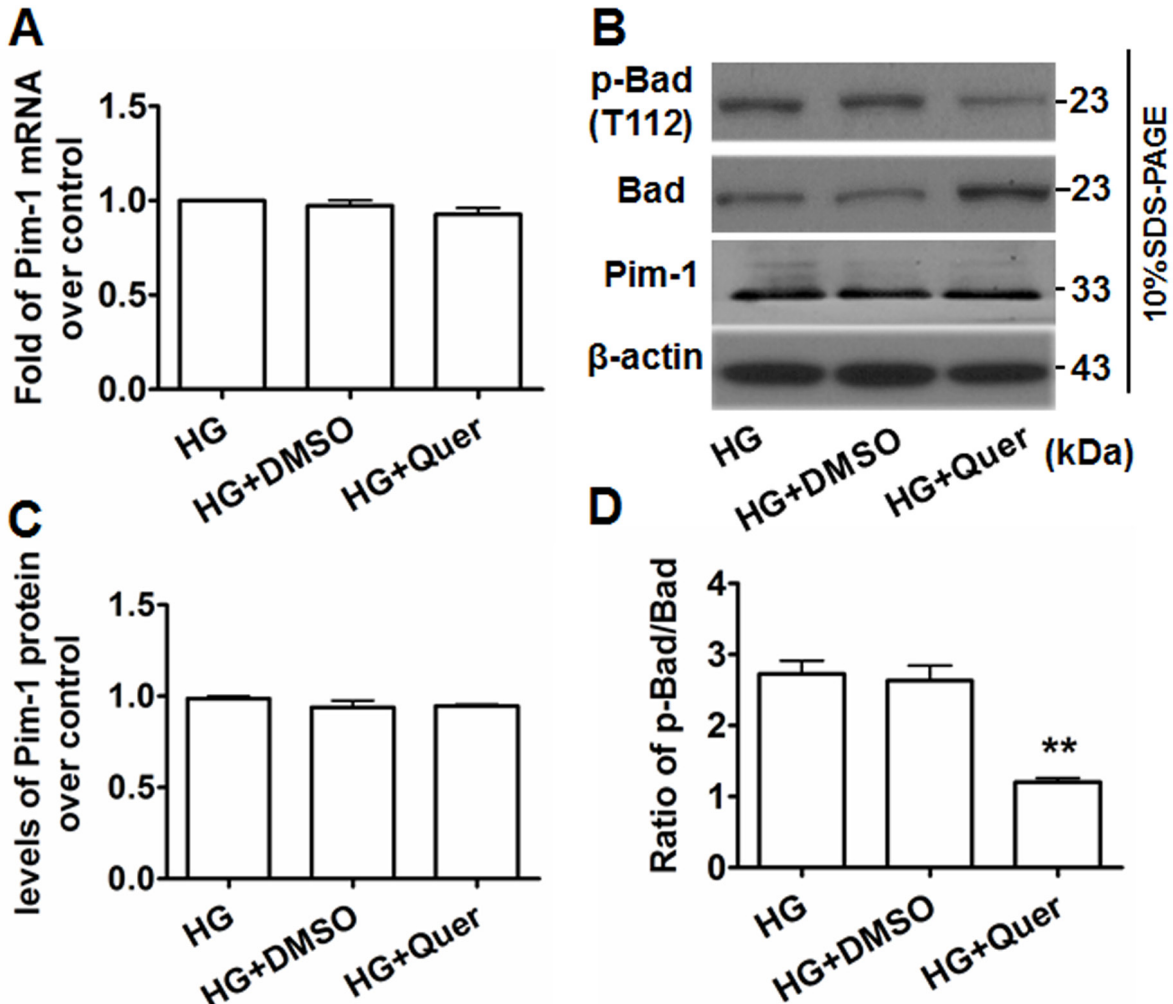
Quercetagetin inhibits Pim-1 activity but not its expression in HG-treated VSMCs **A.** qRT-PCR was used to detect Pim-1 mRNA expression in HG-treated VSMCs. **B.** Western blotting was used to detect Pim-1, Bad and p-Bad(T112) protein expression in HG-treated VSMCs. For qRT-PCR and western blotting, VSMCs were maintained in media supplemented with HG (25 mmol/L D-glucose), HG+Quer (25 mmol/L D-Glucose + 5.5 μmol/L quercetagetin), or HG+DMSO (25 mmol/L D-Glucose + 0.3% (v/v) DMSO) for 48 hours, then cells were harvested and subjected to analysis. **C.** Levels of Pim-1 protein in HG-treated VSMCs. **D.** Ratio of p-Bad/Bad in HG-treated VSMCs. **P < 0.01 *vs*. HG or HG+DMSO.

After treating HG-exposed VSMCs with quercetagetin (5.5 μmol/L) for 48 hours, significant decreases of Pim-1 mRNA and protein expression were not observed (Figure 5A-5C), implying quercetagetin has no inhibitory effect on Pim-1 expression in VSMCs. We therefore detected the changes of Pim-1 protein activity. Because Pim-1 kinase promotes inactivation of Bad protein by its phosphorylation on Ser112 [18, 21], modulation of the level of p-Bad at Ser112 implies a change of Pim-1 kinase activity. As shown in Figures 5B and 5D, exposure of VSMCs to quercetagetin (5.5 μmol/L) increased Bad expression but decreased the level of p-Bad(T112). However, the ratio of p-Bad(T112)/Bad was decreased compared with the controls. Bad promotes apoptosis while p-Bad(T112) has an anti-apoptosis effect; consequently the decrease in ratio of p-Bad (T112)/Bad in quercetagetin-treated cells implies an inhibitory effect of quercetagetin on VSMC proliferation. These outcomes indicate that administration of quercetagetin decreased Pim-1 activity but not its expression in HG-treated VSMCs.

### HG induces VSMC proliferation via activation of STAT3/Pim-1 signaling

**Figure 6:**
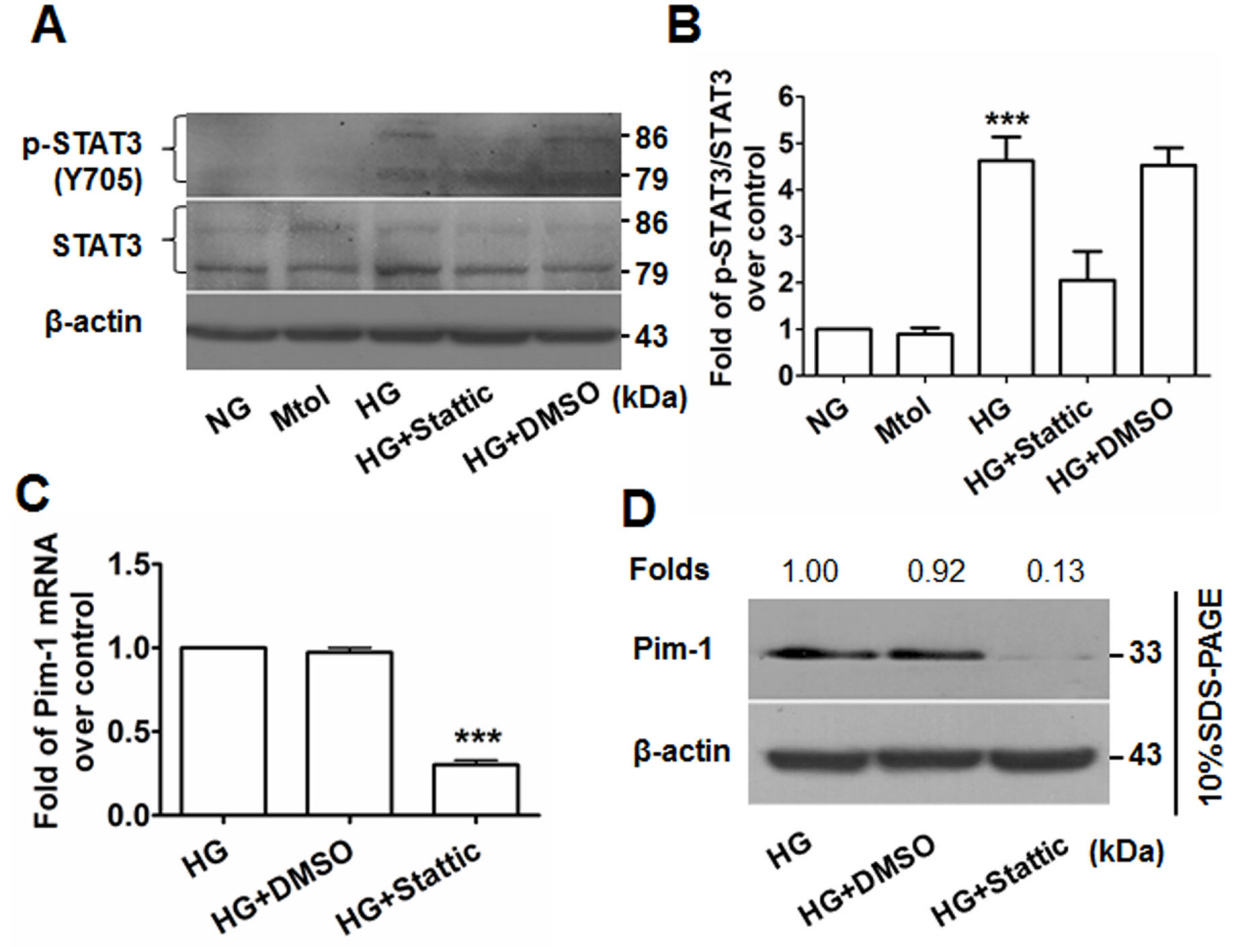
Stattic blocks STAT3 activation and results in attenuated Pim-1 expression in HG-treated VSMCs **A.** p-STAT3(Y705) and STAT3 expression in VSMCs were detected by western blotting. **B.** Ratio of p-STAT3(Y705)/STAT3. ***P < 0.001 vs. NG, Mtol, or HG+Stattic. **C.** qRT-PCR and **D.** western blotting were used to examine Pim-1 mRNA and protein expression in HG-treated VSMCs, respectively. ***P < 0.001 vs. HG or HG+DMSO.

Western blotting showed that upregulation of p-STAT3(Y705) was observed in the HG-treated but not the NG- or Mtol-treated VSMCs, and that administration of stattic (10 μmol/L) decreased the levels of p-STAT3(Y705) (Figures 6A & 6B). Consequently, attenuated expression of translational Pim-1 and PCNA were observed (Figures 6C & 6D, Figure 3D). These results showed that HG induced VSMC proliferation partly through HG-activated STAT3/Pim-1 signaling.

## DISCUSSION

Excessive proliferation of VSMCs derived from the tunica media is a major factor involved in DM-induced atherosclerosis. Moreover, the factors involved in VSMC proliferation are complex and unsolved. We have previously proven that HG induces VSMC proliferation and migration via SDF1/CXCR4/PI3K/Akt signaling [25]. Consistent with our previous study, we report here that STZ-induced HG led to significantly increased proliferation of rat aortic VSMCs *in vivo*. This was evidenced by the upregulation of PCNA expression, with *in vitro* HG stimulation resulting in a significant increase of VSMC growth, as indicated by the cell viability, cell number and PCNA expression. Furthermore, HG stimuli altered the expression of contractile and synthetic markers of VSMCs. Changes in expression of the contractile and synthetic markers of VSMCs support the phenotypic changes of VSMCs under HG conditions [29], which implying one of the reasons for DM-induced atherosclerosis. Interestingly, upregulation of Pim-1 expression was observed in both STZ-induced diabetes and HG-treated VSMCs. Besides, we revealed that HG enhanced both 33-kDa and 44-kDa Pim-1 isoform in VSMCs in a time-dependent manner, which indicated HG condition not only favors VSMCs proliferation but also inhibits apoptosis. Moreover, HG condition prominently favored the 33-kDa but not the 44-kDa isoform. In addition, 25 mmol/L D-glucose was optimal for Pim-1 expression. Interestingly, the Pim-1 inhibitor quercetagetin attenuated HG-induced VSMC proliferation. These findings are consistent with previous reports that Pim-1 is expressed in the VSMCs of balloon-injured rat carotid arteries and human coronary arteries [17], and in the VSMCs of PAH patients [19]. To the best of our knowledge, the present study provides details regarding aberrant Pim-1 expression in HG-insulted VSMCs.

Pim-1 contributes mainly to cell cycle progression and anti-apoptotic signaling. Reports have documented that Pim-1 kinases are phosphorylated and down-regulated by p21/27Kip1 at transcriptional and posttranscriptional levels [30, 31]. Cdc25A/C, a direct transcriptional target for c-Myc, physically interacts with and is phosphorylated by Pim-1 [32]. Furthermore, Pim-1 promotes formation between NuMA, HP1beta, dynein, and dynactin, a complex that is necessary for mitosis [33]. The transcriptional co-activator p100, which interacts with the c-Myb transcription factor, binds to and is phosphorylated by Pim-1 [34]. Moreover, it has been shown that Pim-1 plays a pivotal role in the regulation of survival signaling through modulation of Bcl-2 family members [35]. As a highly selective Pim-1 inhibitor, quercetagetin interacts with the ATP binding site of the Pim-1 protein, leading to impaired Pim-1 activity but not impaired expression. The outcomes of the present investigation show that administration of HG-treated VSMCs with quercetagetin resulted in changes in the ratio of p-Bad(T112)/Bad but not Pim-1 expression. Consequently, attenuated HG-induced VSMC proliferation and cell cycle progression occurred. Our results are consistent with other reports in that treatment with Pim-1 inhibitors effectively induces cell cycle arrest [36-38], and administration of quercetagetin or Pim-1 siRNA inhibits PDGFbb-induced VSMC proliferation [18].

Pim-1 kinase is constitutively active when expressed and its regulation occurs primarily *via* transcription and protein stabilization [39]. Many mitogenic stimuli including growth factors such as interleukins, GM-CSF and G-CSF, and interferon regulate Pim-1 expression at transcriptional level [40]. The majority of these factors transduce their primary signals through the JAK/STAT pathway, indicating that this cascade is essential for regulating the expression of Pim genes [41], which is one of reason why we focused on STAT signaling presently. As a member of the STAT family, STAT3 was originally identified as an acute-phase response factor. It is reported that STAT3 binds to the Pim-1 promoter at the ISFR/GAS-sequence (IFN-γ activation sequence), implying direct regulation of Pim-1 expression [42]. Paulin et al. [19] have reported that STAT3/Pim-1 activation promotes pulmonary artery smooth muscle cell proliferation. In our present study, HG stimuli activated STAT3, evidenced by the upregulation of p-STAT3(Y705). Moreover, administration of stattic, an inhibitor of STAT3, significantly attenuated HG-induced STAT3 activation and VSMC proliferation. Interestingly, stattic-inhibited STAT3 activation led to a down-regulation of Pim-1 expression. Thus, the present study suggests that HG-induced VSMC proliferation might partly be due to the activation of STAT3/Pim-1 signaling, consistent with other reports [19, 43, 44].

Previous reports have indicated that Pim-1 is down-regulated in cardiomyocytes of SZT-induced hyperglycemic rats, especially in females [45]. On the contrary, forced expression of Pim-1 would be beneficial for curing diabetic cardiomyopathy [46]. Recently, a report also indicated HG suppressed Pim-1 expression in vascular endothelial cells (EPCs) [47]. However, our current study indicated that HG conditions induced Pim-1 expression in VSMCs at the manner of transient decline following sustained increase. Because HG condition favors VSMCs proliferation while attenuates EPCs viability [48, 49], it seems that VSMCs displays various responses post-HG insults when compared with EPCs and cardiomyocytes, the underlying mechanisms remain to be further elucidated. Report [45] also indicated Pim-1 was downregulated in *ex vivo* aortic rings of diabetic mice, which is inconsistent with our current results. The reasons are unknown. EPCs also participate in the pathological process of DM-induced vascular remodeling; we just focused on VSMCs in the current investigation. Because the complex role of HG in Pim-1 expression in VSMCs and EPCs, we need another model to interpret the interaction between VSMCs and EPCs in HG-induced vascular remodeling process in future.

Taken together, the present study provides the first evidence that HG treatment can induce VSMC proliferation, which is partly mediated by HG-activated STAT3/Pim-1 signaling. Therefore, Pim-1 overexpression plays a pivotal role in DM-induced vascular complications. Our study provides novel insight regarding DM-related vascular remolding diseases.

## MATERIALS AND METHODS

### Establishing a rat model of type I diabetes

Male Sprague Dawley (SD) rats weighing 250 ± 50 g were purchased from the Center of Experimental Animals of Guangdong Medical University, Zhanjiang, China. All procedures were performed in accordance with the Guidelines of the Guangdong Council of Animal Care and approved by the Animal Use Subcommittee at Guangdong Medical University. Diabetes was induced in rats by single intraperitoneal injection of STZ (55 mg⁄kg, Sigma-Aldrich, Shanghai, China) as previously described [25]. Blood glucose was monitored for up to 3 weeks, and only rats with blood glucose levels >/= 16.6 mmol/L were used in the study. Three weeks after STZ injection, rats were sacrificed and aortic arch, thoracic and abdominal arterial samples were obtained. The tunica media was quickly immersed in liquid nitrogen for later use; for paraffin-embedded sections, samples were fixed in neutral formalin.

### Cell isolation and culture

Primary VSMCs were isolated from SD *rat* aortic arteries and identified as described previously [25]. VSMCs were maintained in Dulbecco’s Modified Eagle Medium (DMEM, HyClone™, GE Healthcare Life Sciences, Utah, USA) containing 5.5 mmol/L glucose and 10% fetal bovine serum (FBS, HyClone™). All cells were maintained at 37°C in a 5% CO_2_ atmosphere. DMEM containing 12.5, 25 and 50 mmol/L D-glucose was used as HG in all experiments, while DMEM containing 5.5 mmol/L glucose (normal glucose, NG) and DMEM containing 25 mmol/L D-mannitol (Mtol) were used as the NG control and osmotic control, respectively. Cells at passages 4-6 were used for experimentation. In all experiments, cells were serum-starved for 12 hours with DMEM containing 0.5% FBS and then subjected to the different treatments.

### Determining cell viability and cell number

*In vitro* cell proliferation assays were performed as described previously [50, 51]. Briefly, VSMCs (2 × 10^3^/well) were seeded into 96-well plates (Corning, Lowell, MA, USA). Cells were cultured in NG, Mtol and HG media supplemented with quercetagetin (5.5 μmol/L, Pim-1 inhibitor, Merck, Germany), stattic (10 μmol/L, STAT3 inhibitor, Merck) and dimethylsulphoxide (DMSO, ≤0.3%, v/v) for 48 hours. After washing twice with 1×PBS, a total of 100 μL DMEM plus 10 μL CCK-8 reagents (Beyotime Institute of Biotechnology, Jiangsu, China) were added to each well and the optical density (OD) was measured 4 hours later using a microplate reader (Multiskan MKS, Thermo Scientific, MA, USA) at dual wavelength (450/630 nm). Each group was duplicated in six wells and each assay was performed in triplicate.

Cell number was determined as described previously [52]. Briefly, VSMCs were seeded into 6-well plates (5 × 10^5^ per well). After overnight recovery in an incubator at 37°C/5% CO_2_, the cells were serum-deprived for 12 hours and exposed to the aforementioned conditions for 48 hours and then counted using a hemocytometer ( *n* = 3 wells / group).

### Flow cytometric determination of cell cycles

VSMCs were seeded into 6-well plates and treated as described above (*n* = 3 wells/group). Forty-eight hours later, harvested cells were stained with *propidium iodide* (PI) and were subjected to flow cytometric analysis (BD FACS Canto II).

### RNA extraction, reverse transcription (RT) and quantitative PCR

RNA extraction, RT and quantitative PCR were performed as described previously [53]. Primer pairs (5´-3´) were synthesized by Sangon Biotech Co., Ltd. (Shanghai, China) as follows: Pim-1 (NM_017034.1) forward, ctgctcaaggacacagtctaca; Pim-1 reverse, agggacaggcaccatctaataa; PCNA (NM_022381.1) forward, gggtgaagttttctgcgagt; PCNA reverse, cagtggagtggcttttgtga; β-actin (NM_031144) forward, cccatctatgagggttacgc, β-actin reverse, tttaatgtcacgcacgatttc. PCR was conducted using a LightCycler480 II instrument (Roche Ltd., Guangzhou, China). Relative abundances of target mRNAs were determined from the C_T_ values and plotted as the fold-change compared with the control groups.

### Western blotting

Western blot analyses were performed using protein extracts from VSMCs as previously described [54, 55]. A total of 30 - 75 μg of protein was transferred onto PVDF membranes by electrophoretic transfer following electrophoretic separation by 10% - 12% SDS-polyacrylamide gel electrophoresis (SDS-PAGE). The antibodies used were Pim-1, PCNA, Bad, p-Bad(T112), STAT3, p-STAT3(Y705), Myocardin, FHL2, Osteopontin, α-SMA and β-actin. Details of these antibodies are included in Supplementary Table S1.

### Immunohistochemistry

Immunohistochemical staining was performed on rat arterial sections. The 5 μM-thick paraffin sections were deparaffinized and re-hydrated according to standard protocols. Heat-induced antigen retrieval was then performed in sodium citrate buffer (10 mmol/L, pH 6.0) and endogenous peroxidases were blocked by incubation in 0.3% H_2_O_2_. Sections were then incubated with primary antibodies against Pim-1 (1:50) and PCNA (1:100) at 4°C overnight and non-immune IgG was used as a negative control. Antigenic sites were localized using the SP9000 or SP9003 kit and 3,3´-diaminobenzidine (DAB) kit (ZSGB-BIO, Beijing, China). Brown coloration in the VSMCs was considered as positive expression.

### Statistical analyses

Statistical analyses were carried out using PRISM Software (GraphPad Software, CA, USA). Data were expressed as means ± standard deviation (SD) from three independent experiments. For analysis of differences between two groups, the Student’s t-test was performed. For multiple groups, analysis of variance (ANOVA) was carried out followed by the Student-Newman-Keuls test. The level of statistical significance was set at *P* < 0.05.

## SUPPLEMENTARY MATERIALS FIGURES AND TABLE


