# Supplementary material for: High glucose promotes vascular smooth muscle cell proliferation by upregulating proto-oncogene serine/threonine-protein kinase Pim-1 expression
Source: Oncotarget. 2017 Jul 18;8(51):88320–31. doi: 10.18632/oncotarget.19368 (PMC5687607; doi:10.18632/oncotarget.19368)
Supplement: Supplementary file 1 [file oncotarget-08-88320-s001.pdf]

High glucose promotes vascular smooth muscle cell proliferation by upregulating proto-oncogene serine/threonine-protein kinase Pim-1 expression

Supplementary MaterialS

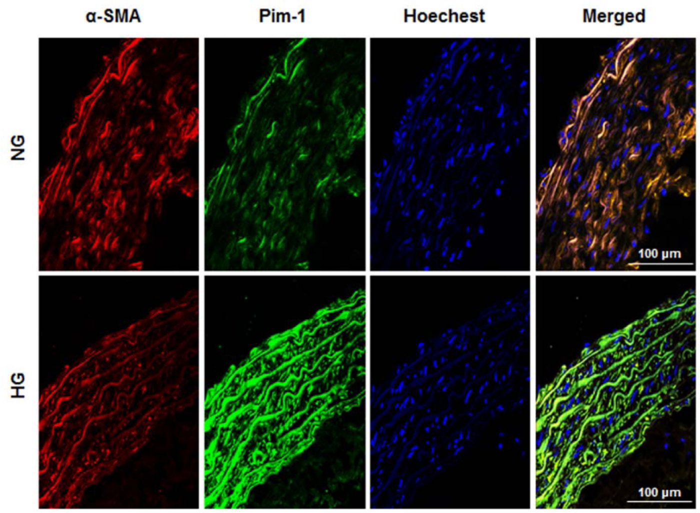

Supplementary Figure S1. Immunofluorescence staining of α-SMA and Pim-1 protein in frozen sections of thoracic artery samples of normolglycemia (NG) and hyperglycemia (HG) rats. Antigenic sites for α-SMA were colorized with PE-coupled IgGs, and the antigenic sites for Pim-1 were colorized with FITC-coupled IgGs, Nuclei were stained with Hoechest332598. Scale bars = 100μm.

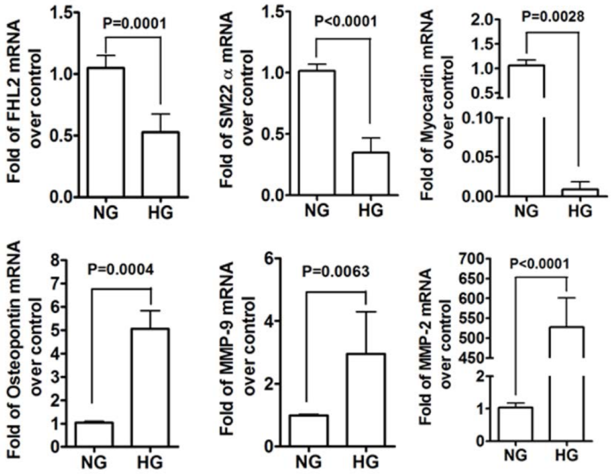

Supplementary Figure S2. The expression of contractile and synthetic markers of VSMC in normolglycemia (NG) and hyperglycemia (HG) rats. Quantitative RT-PCR was used to detect the mRNA levels of contractile markers (FHL2, SM22α, Myocardin) and synthetic markers (Osteoponin, MMP-2, MMP-9) of VSMCs. Data were duplicated as Mean ± SD from three independent experiments. Nor, Normoglycemia; Hyp, Hyperglycemia.

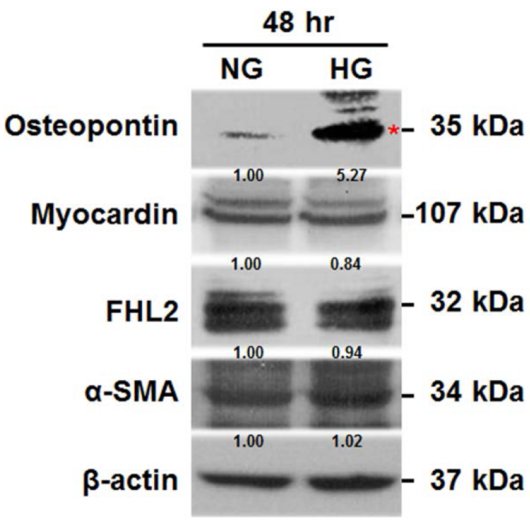

Supplementary Figure S3. Exposure of HG for 48 hours induces the expression of contractile and synthetic markers of VSMC *in vitro*. Western blot analysis was used to examine the expressions of contractile markers (FHL2, α-SMA, Myocardin) and synthetic markers (Osteoponin) of cultured VSMC maintained in high D-glucose condition (25 mmol/L, 48 h). * indicates the upregulated bands.

Supplementary Table S1. Details of antibodies used.

Name	Cat. number	Company	Working dilution	Application
Pim-1	sc-7856	Santa Cruz	1:200	Immunohistochemistry
Pim-1	#2907S	Cell Signaling	1:500	Western blot
PCNA	#2586S	Cell Signaling	1:200<br>1:500	Immunohistochemistry<br>Western blot
p-Bad(T112)	#5284S	Cell Signaling	1:500	Western blot
Bad	#9292S	Cell Signaling	1:500	Western blot
STAT3	bs-1141R	Bioss	1:200	Western blot
p-STAT3(Y705)	sc-7993	Santa Cruz	1:50	Western blot
β-actin	60008-1-Ig	PreteinTech	1:3000	Western blot
Myocardin	M8948	Sigma Aldrich	1:1000	Western blot
α-SMA	sc-53015	Santa Cruz	1:1000	Western blot
Osteopontin	bs-0026R	Bioss	1:500	Western blot
FHL2	bs-5157R	Bioss	1:100	Western blot
